# Physical Indicators of Aging are Related to Cellular Senescence Signal P16INK4a in Midlife Adults

**DOI:** 10.1093/geroni/igab046.3631

**Published:** 2021-12-17

**Authors:** Kelly Rentscher, Teresa Seeman, Steve Cole, Judith Carroll

**Affiliations:** University of California, Los Angeles, Los Angeles, California, United States

## Abstract

Cellular senescence signal p16INK4a has been identified as a biomarker of aging that accumulates with chronological age across several tissues in mice and humans and may be potentially modifiable by interventions. This study examined whether physical indicators of aging were associated with p16INK4a and other markers of the aging process in midlife adults. Participants were 543 adults aged 26–78 years (Mage=54.0; 50.5% female) in the Midlife in the United States Refresher cohort. Interviews, questionnaires, and performance tests measured physical indicators of aging, including the Fried frailty index, limitations in daily activities, and age-related comorbidities. RNA sequencing of whole blood assessed biomarkers of aging: p16INK4a (CDKN2A), the DNA damage response (DDR), and the senescence-associated secretory phenotype (SASP). Older age was associated with enhanced p16INK4a (r=.11, p=.01), DDR (r=.34, p<.001), and SASP (r=.38, p<.001) expression. Multiple regression models that adjusted for age, sex, race/ethnicity, BMI, comorbidities, and time between assessments revealed that frailty (pre-frail/frail vs. non-frail) was associated with greater p16INK4a (B=0.13, p=.048) and marginally greater DDR (B=0.06, p=.06) expression. Limitations in daily activities were also associated with p16INK4a (B=0.12, p=.045). History of heart disease, stroke, arthritis, and cancer were associated with DDR and SASP expression in unadjusted models only (ps<.05). In summary, senescence indicator p16INK4a was elevated in whole blood samples from middle-aged adults who showed signs of frailty and limitations in daily activities. Findings suggest that whole blood p16INK4a expression might potentially be used to detect early signs of aging and target interventions to reduce biological aging and frailty.

